# Editorial: Function and formation of mitochondrial metalloproteome

**DOI:** 10.3389/fcell.2022.1020441

**Published:** 2022-09-28

**Authors:** Michał Wasilewski, Vishal M. Gohil, Oleh Khalimonchuk

**Affiliations:** ^1^ International Institute of Molecular Mechanisms and Machines (IMol), Polish Academy of Sciences, Warsaw, Poland; ^2^ Department of Biochemistry and Biophysics, Texas A&M University, College Station, TX, United States; ^3^ Department of Biochemistry, University of Nebraska, Lincoln, NE, United States; ^4^ Nebraska Redox Biology Center, University of Nebraska, Lincoln, NE, United States; ^5^ Fred & Pamela Buffett Cancer Center, Omaha, NE, United States

**Keywords:** cofactors, copper, heme, iron, metal homeostasis, metalloproteins, mitochondria, transition metals

Mitochondria are essential metabolic and redox hubs of eukaryotic cells, which apart from their best-known role in energy generation, participate in a plethora of biochemical processes. Many of the critical mitochondrial functions depend on transition metals as cofactors of enzymes that are housed within the organelle. One canonical example is the mitochondrial respiratory chain, which utilizes the redox chemistry of iron-sulfur clusters, copper ions, and heme to transport electrons from reducing equivalents such as NADH and FADH_2_ to molecular oxygen, and in the process generate electrochemical potential across the inner mitochondrial membrane that powers ATP synthesis. Extending well beyond the energy conversion function, transition metals are also essential for mitochondrial biosynthetic and proteolytic machinery, and antioxidant defenses. Moreover, mitochondria are the sites of synthesis of vital metal-containing cofactors such as heme and iron-sulfur clusters that support a variety of cellular functions inside and outside of mitochondria. Last but not least, metal homeostasis in mitochondria influences the management of transition metals at the cellular level. Therefore, unraveling the mechanisms behind the function, formation, and regulation of mitochondrial metalloproteome is crucial to understanding the involvement of transition metals in the life of eukaryotic cells.

In recent years, various aspects of the role and regulation of transition metals homeostasis in mitochondria were explored. An important research hotspot was the assembly of metal cofactors into mitochondrial metalloproteins and the machinery that chaperones and secures safe handling of immature cofactors. Another vital development includes the identification of key enzymes that catalyze the synthesis of metal cofactors such as iron-sulfur clusters and heme and proteins involved in the delivery of copper to the respiratory complex IV, also known as cytochrome *c* oxidase. Nonetheless, a number of outstanding questions in the area of mitochondrial transition metal homeostasis remain to be addressed. For example, the regulation of metal homeostasis in mitochondria and coordination of cofactor biosynthesis with other functions of these organelles remains to be elucidated.

This Research Topic of Frontiers in Cellular and Developmental Biology features three reviews and one original research report that provide state-of-the-art perspectives on the biogenesis of mitochondrial metalloproteome, its functions, and regulation.

The review by Medlock et al. for the first time summarizes and discusses an intriguing connection between homeostasis of mitochondrial transition metals and mitochondria contact site and cristae organizing complex that emerged over the last few years. The unique (and still largely uncharted) spatial organization of mitochondrial membranes is inherently intertwined with metabolic and energy transitions in mitochondria. Could it also serve as a coupling mechanism that efficiently distributes organellar resources to secure an adequate supply of metal cofactors? The review by Yien and Perfetto provides an updated overview of mitochondrial heme synthesis with particular emphasis on mechanisms that regulate this pathway. The authors focus equally on the enzymes directly involved in successive steps of heme synthesis as well as on much less explored mitochondrial transporters that control the movement of heme and critical intermediates across the mitochondrial membranes. In their perspective, the authors underscore the importance of tissue-specific mechanisms that tailor heme synthesis to the requirements of a particular cell type. The authors also stress the significance of protein-protein interactions and complex formation in crucial nodes of this vital pathway. An emerging concept of spatiotemporal regulation of mitochondrial processes is also relevant and applicable to metal homeostasis in the organelle. In the era of cryo-EM technology-powered structural studies that are proven to be so effective in deepening our understanding of the dynamic nature of the other mitochondrial supramolecular machinery, the fact that enzymes involved in heme synthesis form such assemblies is particularly intriguing. Related to that note, the article by Obi et al. presents a comprehensive review of the structure of the key heme biosynthetic enzyme ferrochelatase. Based on the structures of substrate-bound and free ferrochelatase from humans and other species, the authors propose an unifying model of the catalytic cycle of this enzyme that describes mechanisms of entry of protoporphyrin IX and iron into the active site as well as the release of the heme molecule. Obi et al. further discuss regulation and posttranslational modifications of ferrochelatases. Interestingly, the authors review the inhibition of human ferrochelatase as a side effect of certain approved drugs, additionally touching upon the curative potential of the evoked photosensitivity in photodynamic therapies. Finally, the original research paper by Brischigliaro et al. investigates the effects of cytochrome *c* oxidase deficiency in flies and reports on the discovery of new intriguing connections between the fitness of mitochondrial respiratory chain and cellular homeostasis of transition metals such as copper.

Altogether, this Research Topic offers a selection of articles providing comprehensive summaries or conveying new findings pertaining to a complex topic of the biogenesis and maintenance of mitochondrial metalloproteome. Both established and new lines of evidence link these processes to diseases in humans for which no effective treatments currently exist. It is therefore important to understand these facets of mitochondrial biology in order to develop potential new avenues for therapies targeting relevant pathways. We anticipate that this Research Topic will be of equal interest to both the experts in the field and researchers looking to learn more about metals in mitochondria.

